# Commentary: Don’t Spurn the SPRPN

**DOI:** 10.1097/AS9.0000000000000031

**Published:** 2021-01-19

**Authors:** Peter Fagenholz

**Affiliations:** 1 From the Massachusetts General Hospital, Boston, MA.

The authors report the use of a single-incision laparoscopic system (SILS) for debridement of peripancreatic necrosis using a technique they call single-port retroperitoneal pancreatic necrosectomy (SPRPN). This is one of many techniques for debriding pancreatic and peripancreatic necrosis via video-assisted minimally invasive means, a variety of which are referenced by the authors. As minimally invasive techniques and variations proliferate, it is important for readers to remain anchored to the basic principles of the step-up approach, namely delay of intervention to allow for demarcation of necrosis; initial treatment with drainage; and reservation of necrosectomy for patients who fail to improve after these initial measures.

Once necrosectomy is undertaken, each technique has various pros and cons. The SPRPN technique described here utilizes an intermediate-sized incision—larger than dilational techniques which typically use 30 Fr access ports, but smaller than usually employed for “open” video assisted techniques such as video assisted retroperitoneal debridement which usually requires at least a 5-cm incision. Five of the 7 patients in this series were treated with a single SPRPN necrosectomy—one required a second SPRPN, and one underwent conversion to an open procedure for treatment of colonic necrosis. The authors, who have extensive experience with minimally invasive necrosectomy, imply that the visualization and angulated instrumentation afforded by the SILS platform may reduce the total number of debridement procedures required. Although the numbers from this small series cannot definitively prove this, it is a possible strength. This technique may occupy a sweet spot between dilational techniques with very small incisions that typically require multiple reinterventions and more invasive techniques required to achieve a higher rate of complete debridement with a single procedure. Another potential advantage of the technique is familiarity to surgeons. Although in this series the debridement was conducted under continuous irrigation with a nephroscope, it is also possible to insufflate the cavity with carbon dioxide and use standard single-port laparoscopic instruments, which may feel more comfortable to surgeons and could potentially increase uptake. SILS ports typically have a length/depth of 6 cm, and the deployment of the instruments within the cavity requires some working space and so this technique will primarily be applicable to medium to large-sized collections tracking to the abdominal wall in the flank or paracolic space, as was the case in this series.

The long hospital stays and the morbidity seen in this series serve to illustrate that no necrosectomy technique will be a panacea for the treatment of infected necrosis, nor should we expect one to be. Matching the optimal technique to each patient based on the anatomy of their necrosis and their overall physiology is one key to achieve efficient necrosectomy and speeding recovery. However, even when this is done to perfection clinicians caring for patients with severe pancreatitis still need to be committed to complex longitudinal care and the treatment of what, for now, are unavoidable complications. I applaud the authors for continuing to meet this challenge and advance the field as they have for years.

